# Survivin −31G>C Polymorphism and Gastrointestinal Tract Cancer Risk: A Meta-Analysis

**DOI:** 10.1371/journal.pone.0054081

**Published:** 2013-02-06

**Authors:** Yan Liu, Lin Li, Haiyan Qi, Yan Gao, Sha Liu, Chongan Xu

**Affiliations:** Department of Oncology, The Fourth Affiliated Hospital of China Medical University, Liaoning, Shenyang, China; Sanjay Gandhi Medical Institute, India

## Abstract

**Background:**

Emerging evidence showed that common functional −31G>C polymorphism (rs9904341 G>C) in the promoter region of the survivin gene is involved in the regulation of survivin expression, thus increasing an individual’s susceptibility to gastrointestinal tract (GIT) cancer; but individually published results are inconclusive. The aim of this systematic review and meta-analysis was to derive a more precise estimation of the association between survivin −31G>C polymorphism and GIT cancer risk.

**Methods:**

A literature search of PubMed, Embase, Web of Science and CBM databases was conducted from inception through July 1st, 2012. Crude odds ratios (ORs) with 95% confidence intervals (CIs) were used to assess the strength of association.

**Results:**

Nine case-control studies were included with a total of 2,231 GIT cancer cases and 2,287 healthy controls. The results indicated that survivin −31G>C polymorphism was associated with increased risk of GIT cancer. In the stratified analysis by cancer types, significant associations were observed between survivin −31G>C polymorphism and increased risk of colorectal and gastric cancers. However, the lack of association of survivin −31G>C polymorphism with esophageal cancer risk may be due to a lack of a sufficient number of eligible studies and the influence of different genetic and environmental factors.

**Conclusion:**

Results from the current meta-analysis suggests that survivin −31G>C polymorphism might increase the risk of GIT cancer, especially among gastric and colorectal cancers.

## Introduction

Gastrointestinal tract (GIT) cancer, especially gastric, esophageal and colorectal cancers, are a global epidemiological health concern [1]. There were estimated 1,500,000 new cases of GIT cancer worldwide in 2005 and the number is expected to rise to 2,110,000 in 2025 [2]. Gastric and colon cancers were the second and third most common causes of cancer-related mortality worldwide in 2008, respectively, accounting for over 1 million deaths [3]. Because early-stage esophageal cancer usually does not express symptoms, it has become an aggressive tumor with a dismal 5-year overall survival rate of under 15% [3]. Generally, GIT cancer is known to be a multifactorial disease induced by complex interactions between environmental and genetic factors [4]. Previous studies suggest that lifestyle, dietary and other environmental exposures, and genetic factors might have played a role in causing GIT cancer [5]. However, the majority of genetic variants that influence susceptibility to GIT cancer are still not well-known [6]. Genetic factors may be important contributors to the risk of GIT cancer. Uptill now, a wide range of gastrointestinal cancer susceptibility genes have been identified, including NAT1/2, GSTM1, CYP2E1, p53, XRCC1, cyclinD1, IL-1, MMP2, survivin, etc [7]–[10]. Mutations in these candidate genes have already been linked to elevated risks of developing GIT cancers [11], [12].

Survivin, an inhibitor of apoptosis protein (IAP), is involved in the regulation of apoptosis and in cell cycle control [13]. The human survivin gene, located on chromosome 17q25, is approximately 14.7 kbp and consists of 4 exons and 3 introns [14]. Various clinical and experimental studies have shown that increased expression of survivin plays an important role in the development and progression of malignant neoplasms by reducing tumor cell apoptosis [15]. Therefore, survivin can be used as a a biomarker and a primary chemotherapeutic target for the detection and treatment of GIT cancer, including esophageal, gastric, and colorectal cancers [16]–[19]. Different genetic variations located in the regulatory regions of the survivin gene have also been discovered to attribute to the over-expression of survivin.

More than 10 common single nucleotide polymorphisms (SNPs) in the promoter region of the survivin gene have been reported, but the −31G>C polymorphism (rs9904341 G>C) is one of the most common variants. The survivin −31G>C polymorphism is a transversion mutation of G to C substitution at position −31 in the promoter region [20]. Recently, many studies have investigated the role of the survivin −31G>C polymorphism in gastrointestinal cancers. Most of the studies support the mechanism in which the expression of survivin gene promotes tumor development and progression by inhibiting apoptosis and increasing cell proliferation [15]. Over-expression of survivin gene has been associated with shorter survival time and poor prognosis in malignancies [19], [21]–[23]. However, there are also some studies suggesting that there exists no association between survivin gene expression and its effects on susceptibility to gastrointestinal cancers [24], [25]. The controversial results are probably due to the differences in the baseline characteristics of patients, including age, morphologic and histological type, differentiation, disease stage, ethnicity, etc [26]. Two recent meta-analyses by Srivastava et al and Wang et al have shown that the survivin −31G>C polymorphism might be associated with an increased risk of cancer, especially among Asian populations [26], [27]. However, they failed to observe increased risks of gastric and esophageal cancers. There are three main reasons for their negative results. Firstly, a gastric study [28] and two colorectal studies [29], [30] were not searched and included by the two meta-analyses, which results in their relatively small sample size. Secondly, in these meta-analyses, the authors performed subgroup analyses based on ethnicity and cancer types in exploring sources of heterogeneity. Numerous other factors, however, may also have caused the observed heterogeneity, such as differences in genotype methods, source of controls, countries and regions, Hardy-Weinberg equilibrium (HWE) in controls, etc. Lastly, in the subgroup analysis by cancer type, they only performed further analyses on gastric and esophageal cancers but not on colorectal cancer due to small sample sizes. Our recent meta-analysis is aimed to update previous meta-analyses, as well as to provide a more comprehensive and reliable conclusion on the associations between survivin −31G>C polymorphism and GIT cancer risk.

## Materials and Methods

### Literature Search

Relevant papers published before July 1st, 2012 were identified through a search in PubMed, Embase, Web of Science and CBM databases using the following terms: (“genetic polymorphism” or “polymorphism” or “SNP” or “gene mutation” or “genetic variants”) and (“gastrointestinal tract neoplasms” or “cancer of gastrointestinal tract” or “gastrointestinal tract cancer” or “esophageal neoplasms” or “gastrointestinal stromal tumors” or “intestinal neoplasms” or “stomach neoplasms” or “gastric cancer” or “esophageal cancer” or “colorectal cancer” or “intestinal cancer”) and (“surviving” or “BIRC5 protein, human” or “EPR-1”). The references used in eligible articles or textbooks were also reviewed to find other potentially sources. Disagreements were resolved through discussion between the authors.

### Inclusion and Exclusion Criteria

Studies included in our meta-analysis have to meet the following criteria: (a) case-control study or cohort study focused on associations between survivin −31G>C polymorphism and GIT cancer susceptibility; (b) all patients diagnosed with GIT cancers should be confirmed by pathological or histological examinations; (c) published data about the size of the odds ratio (OR), and their 95% confidence interval (CI) must be sufficient. Studies were excluded when they were: (a) not a case-control study or a cohort study; (b) duplicates of previous publications; (c) based on incomplete data; (d) meta-analyses, letters, reviews or editorial articles. If more than one study by the same author using the same case series was published, either the studies with the largest sample size or the most recently published study was included. The supporting PRISMA checklist is available as supporting information; see Supplement S1.

### Data Extraction

Using a standardized form, data from published studies were extracted independently by two authors. For each study, the following characteristics and numbers were collected: the first author, year of publication, country, language, ethnicity, study design, numbers of subjects, source of cases and controls, pathological type, detecting sample, genotype method, allele and genotype frequencies, and evidence of Hardy-Weinberg equilibrium (HWE) in controls. In case of conflicting evaluations, disagreements were resolved through discussion between the authors.

### Quality Assessment of Included Studies

Two authors independently assessed the quality of papers according to modified STROBE quality score systems [31], [32]. Forty assessment items related to the quality appraisal were used in this meta-analysis with scores ranging from 0 to 40. Scores of 0–20, 20–30 and 30–40 were defined as low, moderate and high quality, respectively. Disagreements were also resolved through discussion between the authors. The supporting modified STROBE quality score systems is available in Supplement S2.

### Statistical Analysis

The strength of the association between survivin −31G>C polymorphism and GIT cancer susceptibility was measured by ORs with 95%CIs under five genetic models, including allele model (C vs. G), dominant model (CC+GC vs. GG), recessive model (CC vs. GG+GC), homozygous model (CC vs. GG), and heterozygous model (CC vs. GC). The statistical significance of the pooled OR was examined by Z test. Between-study variations and heterogeneities were estimated using Cochran’s Q-statistic with a *P*-value <0.05 as statistically significant heterogeneity [33]. We also quantified the effect of heterogeneity by using *I^2^* test (ranges from 0 to 100%), which represents the proportion of inter-study variability that can be contributed to heterogeneity rather than by chance [34]. When a significant Q-test (*P*<0.05) or *I^2^*>50% indicated that heterogeneity among studies existed, the random effects model (DerSimonian Laird method) was conducted for meta-analysis. Otherwise, the fixed effects model (Mantel-Haenszel method) was used. To establish the effect of heterogeneity based on the results from the meta-analyses, we also performed subgroup analysis by cancer types, ethnicity, country, source of controls and genotype methods. We tested whether genotype frequencies of controls were in HWE using the *χ^2^* test. Sensitivity was performed by omitting each study in turn to assess the quality and consistency of the results. Begger’s funnel plots were used to detect publication biases. In addition, Egger’s linear regression test which measures funnel plot asymmetry using a natural logarithm scale of OR was also used to evaluate the publication biases [35]. All the *P* values were two-sided. All analyses were calculated using STATA Version 12.0 software (Stata Corp, College Station, TX).

## Results

### The Characteristics of Included Studies

**Figure 1:**
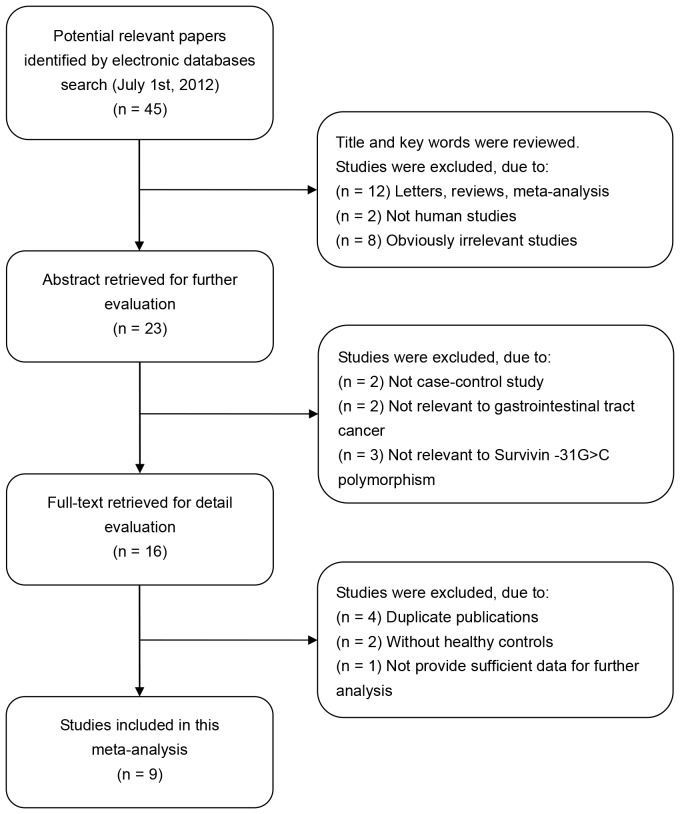
Flow chart of literature search and study selection.

According to the inclusion criteria, 9 studies [21], [22], [24], [25], [28]–[30], [36], [37] were included and 36 were excluded in this meta-analysis. The flow chart of study selection is shown in Figure 1. The total of GIT cancer cases and healthy controls were 2,231 and 2,287, respectively, in these 9 case-control studies. The publication year of involved studies ranged from 2008 to 2011. All patients diagnosed with GIT cancer were also confirmed by pathological examination. Three studies used hospital-based controls, while the other six studies used population-based controls (community populations). All the studies used blood samples for genotyping except for two studies [21], [22] which used tissue samples. A classical polymerase chain reaction-restriction fragment length polymorphism (PCR-RELP) method was performed in seven of the nine studies. Out of the other two studies, one study used Taqman assay and the other used polymerase chain reaction-single strand conformation polymorphism (PCR-SSCP). Overall, there were four gastric cancer studies, three colorectal cancer studies and two esophageal cancer studies. Six of these studies were conducted in Asian populations and three in Caucasian populations. HWE test was conducted on the genotype distribution of the controls in all nine studies. Each study did not deviate from the HWE (all *P*>0.05). All quality scores of included studies were higher than 20 (moderate-high quality). The characteristics of the included studies are summarized in Table 1. The genotype distribution of survivin −31G>C polymorphism is presented in Table 2.

**Table 1:** Characteristics of included studies in this meta-analysis.

First author [Ref]	Year	Country	Ethnicity	Source		Number		Cancertype	Sample	Genotypemethod	Gene	SNP	Aliasname	Qualityscores
				Case	Control	Case	Control							
Cheng et al [21]	2008	China	Asian	HB	PB	96	67	Gastric cancer	Tissue	PCR-RFLP	Survivin	rs9904341 (G/C)	−31G>C	27
Gazouli et al [22]	2009	Greece	Caucasian	HB	PB	312	362	Colorectal cancer	Tissue	PCR-RFLP	Survivin	rs9904341 (G/C)	−31G>C	26
Yang et al-1 [25]	2009	China	Asian	HB	HB	220	220	Gastric cancer	Blood	PCR-RFLP	Survivin	rs9904341 (G/C)	−31G>C	28
Yang et al-2 [37]	2009	China	Asian	HB	PB	221	268	Esophageal cancer	Blood	PCR-RFLP	Survivin	rs9904341 (G/C)	−31G>C	27
Zhu et al [28]	2009	China	Asian	HB	HB	220	220	Gastric cancer	Blood	PCR-RFLP	Survivin	rs9904341 (G/C)	−31G>C	26
Huang et al [30]	2010	China	Asian	HB	PB	702	711	Colorectal cancer	Blood	PCR-RFLP	Survivin	rs9904341 (G/C)	−31G>C	21
Antonacopoulouet al [29]	2010	Greece	Caucasian	HB	PB	163	132	Colorectal cancer	Blood	Taqman	Survivin	rs9904341 (G/C)	−31G>C	27
Upadhyayet al [36]	2011	India	Asian	HB	PB	250	250	Esophageal cancer	Blood	PCR-RFLP	Survivin	rs9904341 (G/C)	−31G>C	27
Borges Bdoet al [24]	2011	Brazil	Caucasian	HB	HB	47	57	Gastric cancer	Blood	PCR-SSCP	Survivin	rs9904341 (G/C)	−31G>C	26

Ref = reference; HB = hospital-based; PB = population-based; PCR-RELP = polymerase chain reaction-restriction fragment length polymorphism; PCR-SSCP = polymerase chain reaction-single strand conformation polymorphism; SNP = single nucleotide polymorphism.

**Table 2:** The genotype distribution of survivin −31G>C polymorphism in case and control groups.

First author [Ref]	Year	Country	Cancer type	SNP	Case							Control							*P* value of HWE test
					Total	G	C	GG	GC	CC	MAF	Total	G	C	GG	GC	CC	MAF	
Cheng et al [21]	2008	China	Gastric cancer	−31G>C	96	78	114	20	38	38	0.59	67	90	44	31	28	8	0.33	0.67
Gazouli et al [22]	2009	Greece	Colorectal cancer	−31G>C	312	267	357	68	131	113	0.57	362	409	315	123	163	76	0.44	0.11
Yang et al-1 [25]	2009	China	Gastric cancer	−31G>C	220	202	238	46	110	64	0.54	220	216	224	47	122	51	0.51	0.10
Yang et al-2 [37]	2009	China	Esophageal cancer	−31G>C	221	218	224	55	108	58	0.51	268	250	286	63	124	81	0.53	0.25
Zhu et al [28]	2009	China	Gastric cancer	−31G>C	220	202	238	46	110	64	0.54	220	216	224	47	122	51	0.51	0.10
Huang et al [30]	2010	China	Colorectal cancer	−31G>C	702	590	814	144	302	256	0.58	711	705	717	180	345	186	0.50	0.43
Antonacopoulou et al [29]	2010	Greece	Colorectal cancer	−31G>C	163	210	116	63	84	16	0.36	132	182	82	66	50	16	0.31	0.18
Upadhyay et al [36]	2011	India	Esophageal cancer	−31G>C	250	302	198	96	110	44	0.40	250	333	167	105	123	22	0.33	0.09
Borges Bdo et al [24]	2011	Brazil	Gastric cancer	−31G>C	47	58	36	20	18	9	0.38	57	70	44	21	28	8	0.39	0.78

Ref = reference; SNP = single nucleotide polymorphism; MAF = minor allele frequency; HWE = Hardy-Weinberg equilibrium.

### Quantitative Data Synthesis

**Figure 2:**
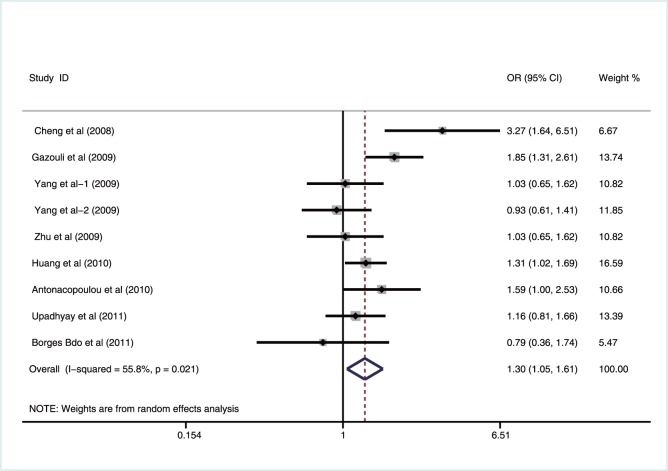
Forest plot of ORs with a random-effects model for associations between survivin −31G>C polymorphism and gastrointestinal tract cancer risk under dominant model (CC+GC vs. GG).

A summary of the meta-analysis findings of the correlation between survivin −31G>C polymorphism and GIT cancer risk is provided in Table 3. The heterogeneity was significant under all genetic models (all *P<*0.05), which might be a result of the difference in cancer types, ethnicity, country, source of controls and genotype methods, so random effects model was used. The meta-analysis results showed that survivin −31G>C polymorphism was associated with increased risk of GIT cancers under all genetic models (allele model: OR = 1.31, 95%CI: 1.10–1.57, *P* = 0.003; dominant model: OR = 1.30, 95%CI: 1.05–1.61, *P* = 0.017; recessive model: OR = 1.54, 95%CI: 1.17–2.03, *P* = 0.002; homozygous model: OR = 1.66, 95%CI: 1.18–2.33, *P* = 0.003; heterozygous model: OR = 1.46, 95%CI: 1.12–1.89, *P* = 0.005) (Figure 2).

**Table 3:** Meta-analysis of the association between survivin −31G>C polymorphism and gastrointestinal tract cancer risk.

Subgroups	C vs. G(Allele model)				CC+GC vs. GG(Dominant model)				CC vs. GG+GC(Recessive model)				CC vs. GG(Homozygous model)				CC vs. GC(Heterozygous model)			
	OR	95%CI	*P*	*P_h_*	OR	95%CI	*P*	*P_h_*	OR	95%CI	*P*	*P_h_*	OR	95%CI	*P*	*P_h_*	OR	95%CI	*P*	*P_h_*
Cancertypes																				
Gastriccancer	1.38	0.92–2.07	0.121†	0.002	1.27	0.75–2.15	0.384†	0.017	1.75	1.07–2.86	0.026†	0.053	1.86	0.90–3.83	0.094†	0.009	1.59	1.14–2.22	0.006†	0.309
Colorectalcancer	1.45	1.20–1.75	<0.001†	0.115	1.51	1.22–1.88	<0.001	0.282	1.58	1.08–2.32	0.020†	0.048	1.84	1.20–2.82	0.006†	0.067	1.38	0.90–2.14	0.144†	0.035
Esophagealcancer	1.08	0.75–1.56	0.673†	0.041	1.06	0.81–1.38	0.696	0.421	1.32	0.50–3.50	0.571†	0.004	1.32	0.51–3.46	0.568†	0.012	1.33	0.50–3.54	0.568†	0.006
Ethnicity																				
Caucasians	1.37	0.99–1.90	0.060†	0.074	1.50	1.01–2.22	0.044	0.153	1.42	0.73–2.79	0.302†	0.051	1.65	0.82–3.32	0.163	0.066	1.26	0.59–2.72	0.549†	0.034
Asians	1.29	1.04–1.61	0.022†	<0.001	1.22	0.95–1.56	0.114†	0.050	1.57	1.12–2.20	0.009†	0.002	1.66	1.09–2.52	0.018†	0.002	1.50	1.11–2.02	0.008†	0.022
Country																				
China	1.30	0.99–1.70	0.056†	<0.001	1.25	0.91–1.71	0.168†	0.027	1.48	1.02–2.15	0.041†	0.002	1.59	0.97–2.58	0.064†	0.001	1.40	1.02–1.93	0.039†	0.028
Greece	1.50	1.07–2.10	0.018†	0.094	1.75	1.33–2.31	<0.001	0.608	1.37	0.52–3.62	0.523†	0.016	1.78	0.71–4.45	0.217†	0.035	1.11	0.37–3.35	0.857†	0.010
India	1.31	1.01–1.69	0.042	–	1.16	0.81–1.66	0.412	–	2.21	1.28–3.82	0.004	–	2.19	1.22–3.91	0.008	–	2.24	1.26–3.97	0.006	–
Brazil	0.99	0.56–1.73	0.965	–	0.79	0.36–1.74	0.553	–	1.45	0.51–4.11	0.484	–	1.18	0.38–3.67	0.773	–	1.75	0.57–5.37	0.328	–
Source ofcontrols																				
Population-based	1.42	1.12–1.81	0.004†	<0.001	1.45	1.12–1.89	0.005†	0.018	1.63	1.09–2.44	0.016†	<0.001	1.89	1.19–3.00	0.007†	<0.001	1.47	1.00–2.15	0.050†	0.001
Hospital-based	1.12	0.94–1.34	0.210	0.898	0.99	0.73–1.33	0.943	0.830	1.37	1.02–1.83	0.035	0.993	1.27	0.88–1.83	0.200	0.991	1.42	1.04–1.92	0.026	0.929
GenotypeMethods																				
PCR-RFLP	1.35	1.10–1.67	0.004†	<0.001	1.31	1.03–1.67	0.029†	0.015	1.64	1.22–2.22	0.001†	0.001	1.79	1.22–2.63	0.003†	<0.001	1.55	1.20–2.00	0.001†	0.024
TaqMan	1.23	0.87–1.73	0.248	–	1.59	1.00–2.53	0.051	–	0.79	0.38–1.65	0.527	–	1.05	0.48–2.27	0.906	–	0.60	0.27–1.29	0.190	–
PCR-SSCP	0.99	0.56–1.73	0.965	–	0.79	0.36–1.74	0.553	–	1.45	0.51–4.11	0.484	–	1.18	0.38–3.67	0.773	–	1.75	0.57–5.37	0.328	–
Overall	1.31	1.10–1.57	0.003†	<0.001	1.30	1.05–1.61	0.017†	0.021	1.54	1.17–2.03	0.002†	0.001	1.66	1.18–2.33	0.003†	0.001	1.46	1.12–1.89	0.005†	0.010

PCR-RELP = polymerase chain reaction-restriction fragment length polymorphism; PCR-SSCP = polymerase chain reaction-single strand conformation polymorphism; OR = odds ratios; 95%CI = 95% confidence interval; P_h_ = P value of heterogeneity test;

† = estimates for random effects model.

**Figure 3:**
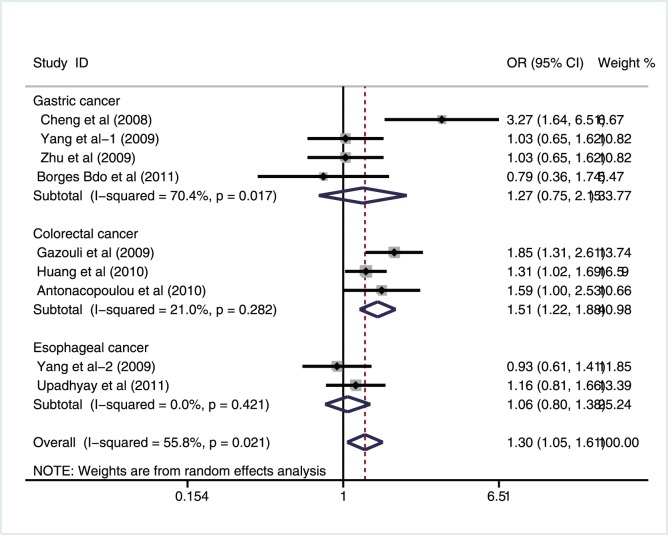
Subgroup analysis by cancer type of ORs with a random-effects model for associations between survivin −31G>C polymorphism and gastrointestinal tract cancer risk under dominant model (CC+GC vs. GG).

In the stratified analysis by cancer types, significant associations were observed between survivin −31G>C polymorphism and increased risk of colorectal cancer under all genetic models (allele model: OR = 1.45, 95%CI: 1.20–1.75, *P<*0.001; dominant model: OR = 1.51, 95%CI: 1.22–1.88, *P<*0.001; recessive model: OR = 1.58, 95%CI: 1.08–2.32, *P = *0.020; homozygous model: OR = 1.84, 95%CI: 1.20–2.82, *P* = 0.006). Furthermore, we also found significant connections between the CC genotype of survivin −31G>C polymorphism and increased risk of gastric cancer under the recessive and heterozygous genetic models (OR = 1.75, 95%CI: 1.07–2.86, *P = *0.026; OR = 1.59, 95%CI: 1.14–2.22, *P* = 0.006; respectively) (Figure 3). However, there was only two studies referred to esophageal cancer susceptibility, which were conducted in India and China [36], [37], respectively. In addition, we also found an obvious difference in the minor allele frequency (MAF) of survivin −31G>C polymorphism in esophageal cancer patients from these two studies (0.40 vs 0.51). Therefore, the lack of association between survivin −31G>C polymorphism and esophageal cancer risk may be due to a lack of a sufficient number of eligible studies and the influence of different genetic and environmental factors.

**Figure 4:**
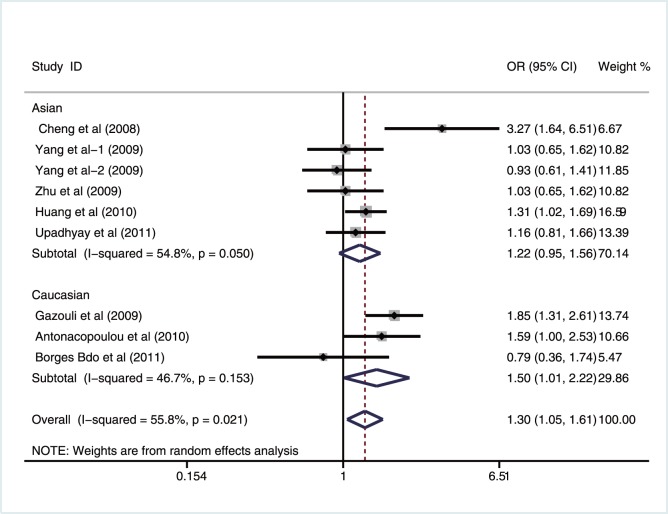
Subgroup analysis by ethnicity of ORs with a random-effects model for associations between survivin −31G>C polymorphism and gastrointestinal tract cancer risk under dominant model (CC+GC vs. GG).

Further stratification analysis by ethnicity, the results showed that survivin −31G>C polymorphism might be a risk factor for GIT cancer among Asian populations under four genetic models (allele model: OR = 1.29, 95%CI: 1.04–1.61, *P = *0.022; recessive model: OR = 1.57, 95%CI: 1.12–2.20, *P = *0.009; homozygous model: OR = 1.66, 95%CI: 1.09–2.52, *P* = 0.018; heterozygous model: OR = 1.50, 95%CI: 1.11–2.02, *P* = 0.008). Also, we found significant associations between the C carrier (CC+GC) of survivin −31G>C polymorphism and increased risk of GIT cancer among Caucasian populations under the dominant model (OR = 1.50, 95%CI: 1.01–2.22, *P* = 0.044) (Figure 4). Subgroup analyses based on country and source of controls, we found that survivin −31G>C polymorphism might increase the risk of gastrointestinal cancer in Chinese, Greek and Indian populations, but not in Brazilian populations. There were also significant associations between survivin −31G>C polymorphism and GIT cancer risk in population-based, hospital-based and PCR-RFLP subgroups (shown in Table 3).

### Sensitivity Analysis

**Figure 5:**
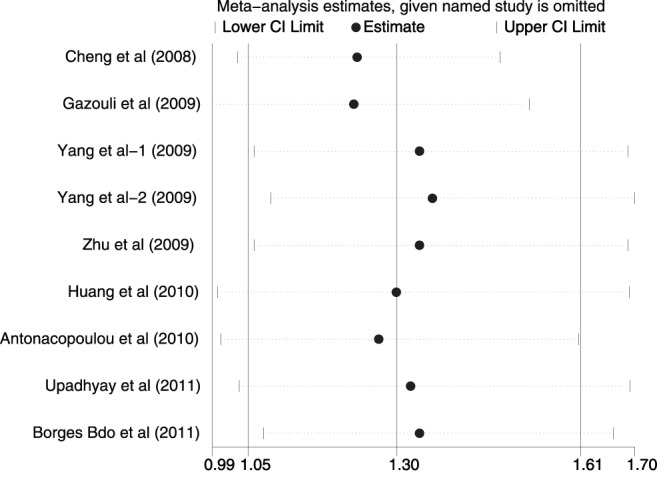
Sensitivity analysis of the summary odds ratio coefficients on the association between survivin −31G>C polymorphism and gastrointestinal tract cancer risk under dominant model (CC+GC vs. GG). Results were computed by omitting each study in turn. Meta-analysis random-effects estimates (exponential form) were used. The two ends of the dotted lines represent the 95% CI.

Sensitivity analysis was performed to assess the influence of each individual study on the pooled ORs by omission of individual studies. The analysis results suggested that no individual studies significantly affected the pooled ORs under any genetic models of survivin −31G>C polymorphism (Figure 5), indicating a statistically robust result.

### Publication Bias

**Figure 6:**
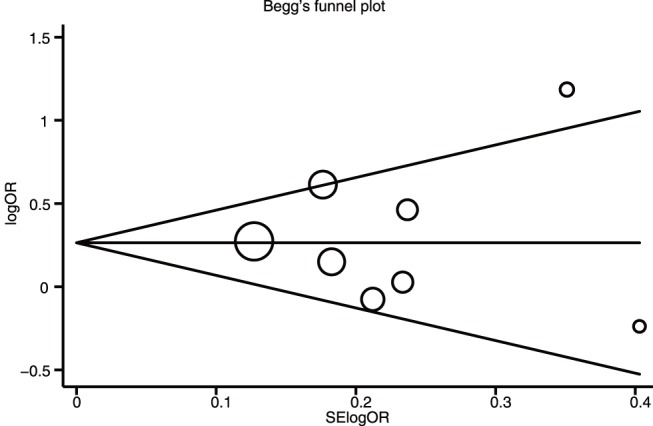
Begger’s funnel plot of publication bias in selection of studies on the survivin −31G>C polymorphism under dominant model (CC+GC vs. GG). Each point represents a separate study for the indicated association. Log[OR], natural logarithm of OR. Horizontal line, mean magnitude of the effect.

Publication biases within available research results might not be representative of all research results. Begger’s funnel plot and Egger’s linear regression test were performed to assess the publication biases of included studies. The shapes of the funnel plots did not reveal any evidence of obvious asymmetry under the dominant model (Figure 6). Egger’s test also showed that there was no strong statistical evidence of publication bias under any genetic models (allele model: t = 0.04, *P* = 0.966; dominant model: t = 0.01, *P* = 0.997; recessive model: t = 0.07, *P* = 0.948; homozygous model: t = 0.03, *P* = 0.974; heterozygous model: t = −0.04, *P* = 0.971).

## Discussion

Survivin, a novel identified member in inhibitor of IAP family, is usually described as an apoptosis inhibitors and plays a key role in anti-apoptosis mechanism of cancer metamorphosis [38]. Unlike other IAPs, survivin is a small protein, has only a single N-terminal baculovirus IAP repeat (BIR) domain and a long C-terminal alpha-helix coiled region, and forms a stable dimmer in solution. The BIR domain is thought to be critical for anti-apoptotic functions with the coiled domain probably interacting with the tubular structures [39]. Survivin inhibits apoptosis via its BIR domain by either directly or indirectly interfering with the functions of caspase-3 and caspase-7 [39]. Abundant studies have suggested that survivin is commonly over-expressed in a wide variety of human malignancies, including lung, breast, stomach, brain, esophagus and liver cancer and is related to clinical progression [40]. Therefore, it is biologically plausible that genetic variations of the survivin gene may modulate cancer risk [41]. The human survivin gene, spanning 14.7 kbp on telemetric position of chromosome 17, contains 4 exons and 3 introns and produces a 16.5-kDa protein [39]. Regulation at the transcriptional level is an important mechanism for survivin expression. Recent findings suggests that a polymorphism located in promoter region (−31G>C) is associated with the alteration of survivin gene expression. This mutation re-upregulates the cell-cycle-dependent transcription of the human survivin gene and results in overexpression of survivin at both mRNA and protein levels [25].

Due to the complex functional mechanism and regulatory roles of survivin in tumorigenesis, the relationships between survivin −31G>C polymorphism and GIT cancer susceptibility have been widely studied, however, these results were inconsistent. A clinical and genetic study suggested that the CC genotype of survivin −31G>C polymorphism might increase colorectal cancer risk among Caucasians [22]. Moreover, similar results have been obtained by Yang et al [25]. Their results suggested that survivin −31G>C polymorphism might be involved in distal gastric carcinogenesis and tumor differentiation among Chinese populations. Nevertheless, previous pathological study has shown that the cytoplasmic survivin expression was not a prognostic factor for advanced esophageal cancer [42]. Two recent meta-analyses by Srivastava et al and Wang et al also indicated that there was no association between survivin −31G>C polymorphism and the risk of gastric and esophageal cancers [26], [27]. However, these meta-analyses did not provide convincing and reliable evidences relevant to survivin −31G>C polymorphism and GIT cancer risk because some relevant case-controlled studies were not included. Furthermore, heterogeneity was clearly evident in the results and could not be explained fully after stratified analyses based on ethnicity and cancer type. In view of these conflicting results from previous studies and the insufficient statistical power of the two recent meta-analyses, we performed the present meta-analysis to update previous meta-analyses and to provide a comprehensive and reliable conclusion by evaluating the association between survivin −31G>C polymorphism and the risk of GIT cancer. In this meta-analysis, including 2,231 GIT cancer cases and 2,287 healthy controls, the results indicated that survivin −31G>C polymorphism was associated with a significantly increased risk of GIT cancer. Although the exact function of survivin in tumorigenesis is not clear yet, a potential explanation might be that survivin gene mutations increased the ability of survivin as an inhibitor of apoptosis and regulator of cell division [43]. In the stratification analysis by cancer types, survivin −31G>C polymorphism showed significant associations with increased risk of colorectal and gastric cancers. As only two eligible studies [36], [37] were identified, we did not find a a statistically significant association between survivin −31G>C polymorphism and esophageal cancer risk. These two studies were conducted in the Indian and Chinese populations from the East Asian region, respectively. However, we found an obviuos difference in the MAF of survivin −31G>C polymorphism in esophageal cancer patients from these two studies (0.40 vs 0.51). Sato et al reported that survivin was highly expressed in esophageal cancer cell lines as compared to normal organ tissues [44]. Several studies have also shown that the expression level of tumor survivin mRNA might be an important prognostic and biological marker regarding esophageal cancer patients [45]–[48]. Rosato et al revealed that survivin expression may be regarded as a prognostic factor only in squamous cell carcinomas but not in adenocarcinomas of the esophagus [49]. Therefore, the lack of association between survivin −31G>C polymorphism and esophageal cancer risk may be due to a lack of a sufficient number of eligible studies and the influence of different genetic and environmental factors. More research is needed to determine the association between survivin gene polymorphisms and esophageal cancer risk. Further stratified analysis by ethnicity and country, the results identified that survivin −31G>C polymorphism as a risk factor for GIT cancer among both Asian and Caucasian populations, and was also associated with increased risk among Chinese, Greek and Indian populations, but not in Brazilian populations. The reasons for the diverse results might include differences in genetic backgrounds and environments, different matching criteria and selection biases.

In interpreting our results of the current meta-analysis, some limitations need to be addressed. Firstly, the sample size is still relatively small and may not provide sufficient statistical power to estimate the correlation between survivin −31G>C polymorphism and GIT cancer risk. Secondly, the selection bias may exist because of the differences in source of controls or detection samples. Thirdly, our meta-analysis was based on unadjusted ORs estimates because not all published presented adjusted ORs and if they did, the ORs were not adjusted by the same potential confounders, such as ethnicity, age, gender, geographic distribution, etc. Nevertheless, it is well acknowledged that many other factors, such as gene-gene or gene-environment interaction may affect the risk of GIT cancer. Furthermore, the present meta-analysis also includes most of the studies from Asian populations, which may not provide strong evidence of heterogeneity by ethnicity. Finally, although all cases and controls of each study were well defined with similar inclusion criteria, there may be other potential factors that were not taken into account that may have influenced our results.

In spite of these limitations, our meta-analysis still has some advantages. The specific aim of this study is to update the previous meta-analyses and focus on the correlation between survivin −31G>C polymorphism and GIT cancer risk. Unlike previous meta-analyses, we find that survivin 31G>C polymorphism is associated with increased risk of gastric and colorectal cancers. It is worthwhile to mention that we have established an effective and efficient searching strategy based on computer-assisted program and manual search to find all possible and eligible studies. Through this search strategy, the quality of studies included in this meta-analysis satisfied our selection criteria. Furthermore, explicit methods for study selection, data extraction, and data analysis were well designed before initiating the calculations. Last but not least, there were no evidences of publication bias in this meta-analysis and the sensitivity analysis indicated that the results are statistically robust.

In summary, this meta-analysis suggests that survivin 31G>C polymorphism may be a risk factor for developing GIT cancer, especially among gastric and colorectal cancers. However, further studies are necessary in order to warrant and validate the associations between survivin gene polymorphisms, other gene polymorphisms and GIT cancer risk.

## Supporting Information

Supplement S1 **PRISMA Checklist.**
(DOC)

Supplement S2 **Modified STROBE quality score systems.**
(DOC)
